# Precision and safety advantages of ultrasound-guided nerve blocks in geriatric anesthesia: current status and future prospects

**DOI:** 10.3389/fmed.2025.1709406

**Published:** 2025-12-17

**Authors:** Xiao-yin Lin, Ya-mei Yang, Yang Liu, Qing-qing Dang, Kan Zhang

**Affiliations:** Department of Anesthesiology, Yan’an People’s Hospital, Yan’an, China

**Keywords:** ultrasound-guided regional anesthesia, nerve block, geriatric anesthesia, postoperative cognitive dysfunction, enhanced recovery after surgery, precision, safety

## Abstract

Ultrasound-guided regional anesthesia (UGRA) has emerged as a pivotal advancement in geriatric anesthesia, significantly enhancing procedural precision and patient safety through dynamic, real-time sonographic visualization. This technology overcomes critical limitations of conventional landmark-based techniques by enabling accurate needle navigation and localized anesthetic delivery, thereby improving block success rates while reducing volume requirements and systemic exposure. In elderly populations, characterized by heightened vulnerability to pharmacological adverse events and perioperative complications, UGRA facilitates opioid-minimized analgesia, attenuates neuroinflammatory responses, and lowers the incidence of delirium and cognitive dysfunction. Its alignment with enhanced recovery after surgery (ERAS) protocols further promotes early mobilization and functional recovery. Despite existing challenges in technical training and resource allocation, ongoing innovations in imaging artificial intelligence, sustained-release local anesthetics, and personalized protocols hold substantial potential to broaden its applications. Future integration of UGRA into perioperative care necessitates standardized competency-based training and rigorously designed multicenter clinical trials to consolidate its role in improving outcomes for the aging surgical population.

## 1 Introduction

### 1.1 The new normal of surgical anesthesia in an aging wave

The global demographic landscape is shifting irreversibly toward an aging population, creating a new normal in surgical practice where elderly patients represent a rapidly expanding cohort (1, 2). This demographic transition poses unique anesthetic challenges due to the confluence of multiple factors: a high prevalence of comorbidities such as cardiovascular disease, diabetes, and renal insufficiency; a progressive decline in physiological functional reserve; and significant alterations in pharmacokinetics and pharmacodynamics (1, 2). These age-related physiological changes collectively heighten the vulnerability of older adults to perioperative complications, demanding a critical reevaluation of standard anesthetic protocols to meet their distinct needs (3, 4).

### 1.2 Limitations of conventional anesthetic techniques

Traditional anesthetic modalities demonstrate considerable shortcomings in this vulnerable population. General anesthesia, while indispensable for many procedures, is associated with a higher incidence of deleterious outcomes in the elderly, including postoperative delirium (POD), postoperative cognitive dysfunction (POCD), prolonged respiratory depression, and hemodynamic instability (4, 5). Similarly, conventional landmark-based or paresthesia-seeking nerve block techniques are hampered by their inherent unpredictability (6–8). These blind approaches often result in inconsistent success rates, necessitate higher volumes of local anesthetic, and carry an inherent risk of complications such as nerve injury, intravascular injection, and systemic local anesthetic toxicity (6, 7). These limitations can impede early mobilization, increase fall risk, and ultimately prolong hospital recovery.

### 1.3 The rise of precision anesthesia and enhanced recovery after surgery (ERAS) philosophy

In response to these challenges, the paradigms of precision anesthesia and ERAS have gained paramount importance (9, 10). These collaborative, evidence-based frameworks advocate for tailored perioperative management strategies designed to minimize physiological stress and accelerate convalescence (9, 10). A core tenet is the implementation of multimodal, opioid-sparing analgesia to facilitate early ambulation and functional recovery (10, 11). Within this modern approach, Ultrasound-guided regional anesthesia (UGRA) has emerged as a transformative technology that enables this essential paradigm shift toward precision medicine (12).

### 1.4 Purpose and framework of this perspective

This perspective study aims to provide a comprehensive critical appraisal of the integral role of UGRA in modern geriatric anesthesia. It will methodically review the current evidence base, analyze the mechanistic foundations for its enhanced safety and efficacy profile, and explore future directions and technological advancements. The objective is to synthesize existing knowledge to inform clinical practice and guide future research. It is expressly stated that this article is a scholarly review and perspective; it does not report original research data but rather offers an informed analysis of the established literature.

## 2 Advantages in precision: the technological revolution from “blind exploration” to “real-time visualization”

The implementation of UGRA constitutes a paradigm shift from dependence on anatomical landmarks and subjective patient feedback to objective, image-guided precision (7, 13). This technological advancement has fundamentally transformed the execution of peripheral nerve blocks, offering unparalleled accuracy that is particularly valuable in elderly patients with altered anatomy and physiology.

### 2.1 Core contributions of visualization technology

The fundamental advancement of UGRA lies in its capacity to provide dynamic anatomical visualization. High-resolution ultrasound imaging enables direct identification of neural structures, surrounding vasculature, needle trajectory, and crucially, the real-time distribution of local anesthetic (14–16). This visual confirmation eliminates dependence on surrogate endpoints such as paresthesia or loss of resistance, which prove particularly unreliable in elderly patients due to frequent pre-existing neuropathies and tissue changes (6, 16). Consequently, UGRA demonstrates significantly improved first-attempt success rates while reducing the number of needle redirections needed (6, 15). The direct visualization of adequate perineural spread provides immediate confirmation of block efficacy, enhancing procedural confidence.

### 2.2 Dose precision and medication minimization

UGRA enables a sophisticated approach to dose optimization through direct visual confirmation of local anesthetic spread. This capability permits the use of significantly reduced volumes and concentrations of local anesthetic while maintaining blockade efficacy (17–19). This precision dosing carries particular importance in geriatric patients (18). The application of lower-concentration solutions provides effective sensory analgesia while preserving motor function, thereby facilitating early mobilization and reducing fall risk following lower extremity blocks (17, 18, 20). Additionally, reducing the total drug mass administered decreases the risk of local anesthetic systemic toxicity—a critical safety consideration in older patients with potentially impaired metabolic function and reduced plasma protein binding (17, 18).

### 2.3 Overcoming anatomical variations

Age-related anatomical changes present substantial challenges to conventional nerve localization techniques (21–23). Common alterations include tissue plane obscuration from loss of fascial integrity, fat infiltration that modifies sonographic appearance, and degenerative skeletal changes that displace traditional landmarks (21–23). UGRA provides the unique capability to dynamically navigate these variations through real-time anatomical assessment (22, 23). The operator can adapt needle positioning based on direct visualization, accurately identifying neural structures despite echogenic adipose tissue and avoiding aberrant vasculature (6, 15, 22). This dynamic adaptation ensures consistent procedural accuracy and enhances patient safety despite anatomical variability.

## 3 Safety advantages: multidimensional enhancement of patient outcomes

UGRA offers a superior safety profile that extends beyond procedural success to encompass comprehensive improvements in perioperative care, particularly for vulnerable geriatric patients (6, 24).

### 3.1 Reduction of systemic complications

UGRA significantly mitigates systemic risks by providing effective regional analgesia that frequently eliminates the necessity for general anesthesia or profound sedation. This approach avoids endotracheal intubation and mechanical ventilation, thereby reducing pulmonary complications such as atelectasis, ventilator-associated pneumonia, and hypoventilation—especially critical for elderly patients with diminished respiratory reserve (25). Furthermore, by delivering targeted pain control, UGRA produces a substantial opioid-sparing effect (26, 27). This reduction in opioid consumption directly decreases the incidence of related adverse effects including postoperative nausea and vomiting, respiratory depression, gastrointestinal dysmotility, and the potential for persistent opioid use (26, 28).

### 3.2 Mitigation of neurological injury risk

While serious neurological injury remains uncommon in regional anesthesia, UGRA enhances procedural safety through continuous visual guidance. Real-time visualization enables precise needle navigation, maintaining a safe distance from neural structures and minimizing the risk of intraneural injection or mechanical trauma (29, 30). The ability to monitor needle trajectory and observe low-resistance injection patterns provides an additional safety mechanism not available in landmark-based techniques, substantially reducing the potential for iatrogenic nerve injury (30–32).

### 3.3 Central nervous system protection

UGRA provides notable neuroprotective benefits through multimodal mechanisms. Excellent regional analgesia attenuates surgical stress response and prevents pain-mediated neuroinflammation, both implicated in cognitive decline (33–35). Importantly, the reduction in opioid administration is particularly valuable for elderly patients, as opioids contribute to delirium through multiple pathways including neuroinflammation, neurotransmitter dysregulation, and sleep architecture disruption (34, 35). By minimizing opioid exposure and maintaining physiological homeostasis, UGRA represents a strategic approach to reducing the incidence and severity of POD and POCD (34, 35).

### 3.4 Facilitation of functional recovery and early discharge

UGRA aligns seamlessly with ERAS protocols by promoting accelerated functional recovery. Targeted analgesic techniques that preserve motor function (e.g., selective sensory blocks) enable early ambulation and participation in rehabilitation activities (36). Maintained motor strength, particularly following lower extremity procedures, reduces fall risk and enhances mobility (37). This combination of effective analgesia, preserved function, and minimized opioid-related complications contributes significantly to reduced hospital length of stay, decreased healthcare costs, and improved patient satisfaction—core objectives in contemporary geriatric perioperative management (38, 39).

### 3.5 Comparative summary of UGRA and traditional anesthetic techniques

A comparative summary of UGRA and traditional landmark-based techniques for elderly patients is presented in Table 1. UGRA demonstrates enhanced procedural precision, which contributes to reduced local anesthetic requirements, diminished systemic toxicity risk, and improved outcomes regarding perioperative neurocognitive disorders. Conversely, the landmark-based approach poses challenges due to anatomical variability and correlates with a higher incidence of complications.

**Table 1:** Comparison between UGRA and traditional landmark-based techniques in elderly patients.

Dimension	UGRA	Traditional techniques
Visualization	Real-time anatomical imaging of nerves, vessels, and needle trajectory	Based on anatomical landmarks and tactile feedback; indirect localization
Precision	High accuracy in needle placement and anesthetic spread	Greater variability; increased failure rate in altered anatomy
Drug dosage	Allows lower anesthetic volumes and concentrations	Requires higher doses; increased systemic exposure
Safety profile	Lower incidence of vascular puncture, nerve injury, and systemic toxicity	Higher risk due to blind needle advancement
Opioid requirement	Significantly reduced; supports opioid-sparing analgesia	Often requires postoperative opioids for adequate pain control
Neuroinflammation & cognitive outcomes	Reduces POD/POCD by minimizing systemic inflammation and opioid-induced central nervous system effects	Greater risk of neuroinflammation and cognitive dysfunction
Functional recovery	Promotes early mobilization and shorter hospital stay	Delayed recovery due to higher pain scores or motor weakness
Training requirement	Requires specialized ultrasound skills and equipment	Easier initial training; limited adaptability in complex cases

UGRA, ultrasound-guided regional anesthesia; POD, postoperative delirium; POCD, postoperative cognitive dysfunction.

### 3.6 UGRA’S multi-pathway reduction of POD/POCD

**Figure 1:**
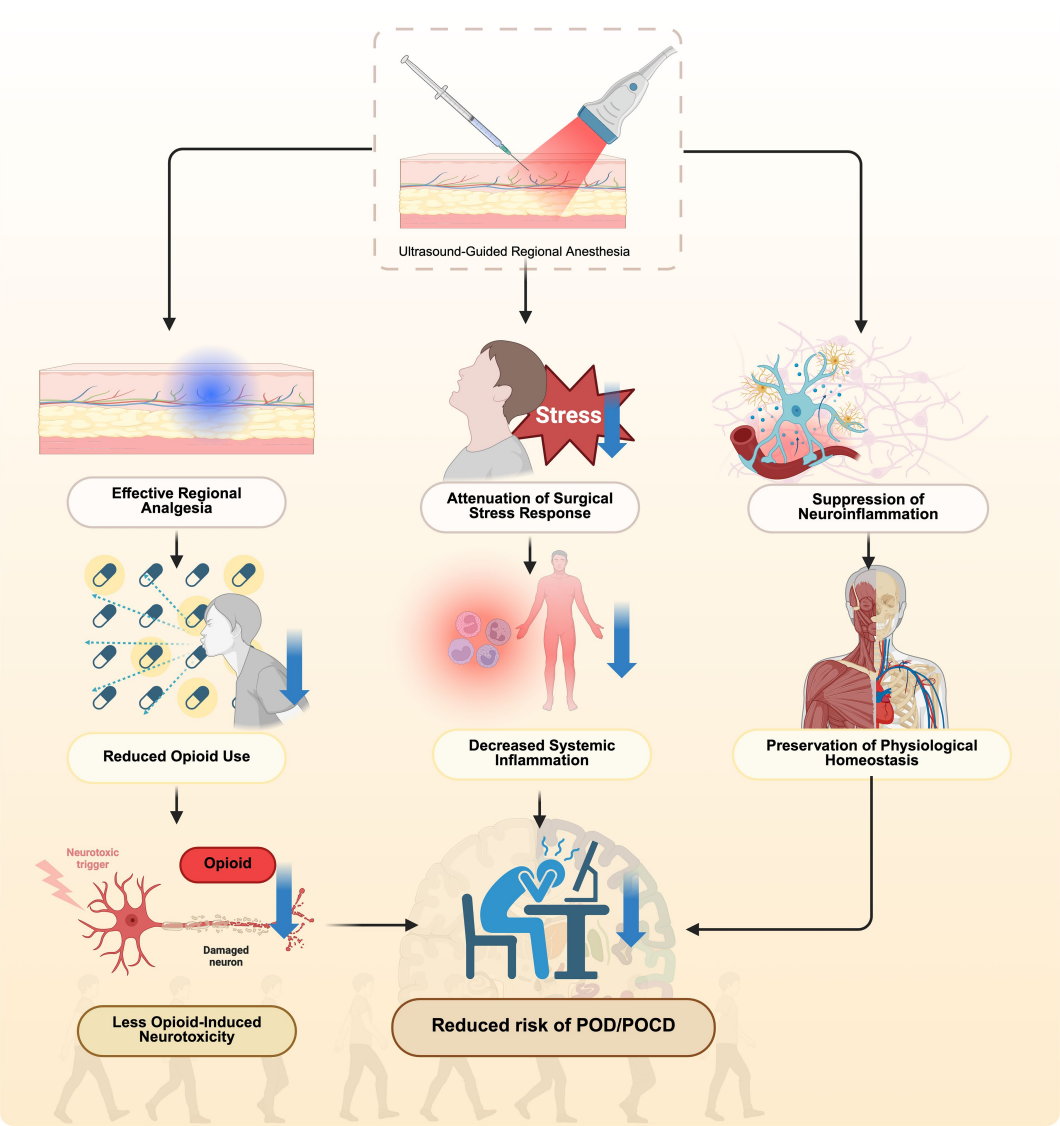
Multimodal mechanisms for UGRA-mediated risk reduction of POD/POCD.

To elucidate the neuroprotective mechanisms underlying UGRA in elderly surgical patients, Figure 1 presents an integrative schematic of its multi-pathway effects on POD and POCD. UGRA provides precise regional analgesia that substantially reduces perioperative opioid requirements, thereby minimizing opioid-induced neurotoxicity, sleep architecture disruption, and neurotransmitter imbalance. In parallel, UGRA attenuates the surgical stress response by modulating autonomic and neuroendocrine activity, leading to decreased systemic inflammatory cytokines such as interleukin-6 and tumor necrosis factor-alpha. Moreover, UGRA mitigates neuroinflammation by preserving blood–brain barrier integrity, limiting microglial activation, and maintaining cerebral homeostasis. Collectively, these coordinated mechanisms converge to preserve neuronal function and cognitive integrity, thereby reducing the overall risk of POD and POCD in the elderly population.

## 4 Current challenges and limitations in clinical application

Notwithstanding its established benefits, the broader adoption and implementation of UGRA encounter several practical and evidence-based challenges that merit critical examination.

### 4.1 Learning curve and disparities in technical dissemination

Achieving clinical proficiency in UGRA necessitates specialized training that extends beyond conventional anesthetic techniques (40). Clinicians must develop competencies in sonographic anatomy interpretation, hand-eye coordination for real-time needle guidance, and the interpretation of dynamic tissue imaging (41). This complex skill acquisition presents a substantial learning curve that requires structured educational programs and supervised clinical experience (40, 41). Significant disparities exist in resource allocation and training opportunities between academic medical centers and community hospitals, creating geographical and institutional inequalities in access to advanced regional anesthesia services (41, 42). Addressing these gaps requires the development of standardized competency-based curricula and increased utilization of simulation-based training modalities.

### 4.2 Application in anatomically challenging populations

Certain patient populations present unique technical challenges for ultrasound-guided approaches. Patients with extreme obesity (class II/III) demonstrate significant ultrasound beam attenuation due to adipose tissue, resulting in diminished image resolution and difficulty visualizing deep neural structures (43). Those with severe spinal deformities (e.g., advanced kyphoscoliosis) or extensive prior surgical interventions may present with fundamentally altered anatomical relationships that complicate standard imaging planes and needle trajectories (44, 45). In such clinical scenarios, adjunct techniques including neurostimulation, alternative transducer positioning strategies, or hybrid approaches may be necessary to maintain procedural safety and efficacy (45–47).

### 4.3 Comprehensive health economic evaluation

The initial capital investment required for high-resolution ultrasound equipment and ongoing maintenance costs represent tangible economic considerations (48). However, a more comprehensive health economic analysis should account for the potential for downstream resource utilization reductions (49). By potentially decreasing complication rates, reducing opioid-related adverse effects, facilitating earlier functional recovery, and shortening hospital length of stay, UGRA may demonstrate favorable cost-effectiveness ratios (49, 50). Future economic evaluations should incorporate long-term outcome measures and total episode-of-care costing to provide more robust evidence regarding its value-based proposition (49).

### 4.4 Critical appraisal of current evidence quality

While the evidence base supporting UGRA continues to expand, it remains essential to acknowledge methodological limitations within the existing literature. Many studies constitute single-center investigations with limited sample sizes, potentially constraining their statistical power and generalizability (51, 52). There persists a need for larger, multi-institutional randomized controlled trials utilizing standardized outcome measures and extended follow-up periods (52, 53). Particularly needed are investigations examining the impact of UGRA on persistent postoperative outcomes including chronic pain development and long-term functional recovery in elderly surgical patients (53).

Future studies must urgently address key methodological challenges to enhance the validity of UGRA research. First, although blinding patients and outcome assessors is feasible, blinding the operator remains difficult due to the procedural nature of UGRA. Therefore, trials should incorporate independent pain management teams and ensure outcome assessors are masked to group allocation to minimize performance and detection bias. Second, standardizing primary outcome measures through consensus is critical. A core outcome set should include postoperative pain scores at rest and during movement, total opioid consumption within defined periods, and long-term cognitive function—such as POCD incidence assessed at 3 months postoperatively using validated neuropsychological batteries. Adopting such standards will enable meaningful cross-study comparisons and data pooling, facilitating a comprehensive evaluation of UGRA’s effects in elderly patients.

## 5 Future directions: technological convergence and conceptual evolution

The ongoing advancement of UGRA is poised to transform perioperative care for elderly patients through the integration of emerging technologies and refined clinical methodologies.

### 5.1 Intelligent system integration

Future progress will center on sophisticated technological integration to augment procedural precision and operational efficiency. Artificial intelligence algorithms capable of real-time anatomical segmentation offer potential for automated nerve identification, optimized needle path planning, and predictive modeling of local anesthetic dispersion (54). For instance, convolutional neural networks can be trained to accurately differentiate neural structures from adjacent tissues in ultrasound imaging, thereby offering real-time, intelligent decision support to clinicians. Such developments could reduce inter-operator variability and enhance procedural standardization (55). Concurrently, augmented reality navigation systems that project processed ultrasound data directly onto the patient’s anatomical field may fundamentally transform spatial orientation during block performance (55). These advanced interfaces promise to diminish the cognitive load associated with traditional ultrasound interpretation and improve technical accessibility.

### 5.2 Advanced pharmaceutical formulations

Innovations in drug delivery systems present new opportunities for extending therapeutic benefits. Sustained-release local anesthetic preparations, particularly liposome-encapsulated formulations, demonstrate potential to provide prolonged analgesia lasting up to 96 h following single administration (56–58). Such extended duration could obviate the need for catheter-based continuous infusion techniques in many clinical scenarios, thereby reducing associated infectious risks and simplifying postoperative care while maintaining effective opioid-sparing analgesia (56, 58).

### 5.3 Personalized protocol development

Future investigations should prioritize the development of tailored analgesic strategies based on individual physiological characteristics. Pharmacokinetic and pharmacodynamic studies establishing optimal drug selection and dosage regimens for patients with age-related comorbidities (e.g., hepatic impairment, cardiac dysfunction) are critically needed (59, 60). Furthermore, rigorous comparative effectiveness research evaluating multimodal adjunct combinations—including *α*-2 agonists, anti-inflammatory agents, and other non-opioid analgesics—will provide evidence-based guidance for optimizing therapeutic ratios and enhancing recovery quality (61, 62).

### 5.4 Expansion of clinical applications

The therapeutic scope of UGRA is likely to expand beyond conventional perioperative boundaries. Its implementation in emergency settings for acute trauma management offers significant potential for improving pain control while avoiding systemic opioid administration in fragile elderly patients (63, 64). Additionally, exploration of its application in chronic pain conditions, particularly neuropathic pain syndromes prevalent in aging populations, represents a promising research direction (65, 66). The integration of ultrasound-guided techniques into palliative care protocols may also provide effective minimally invasive options for managing cancer-related and end-of-life pain, thereby improving overall quality of care (67, 68).

## 6 Summary

UGRA has fundamentally transformed perioperative care for elderly patients by establishing new standards of precision and safety in regional anesthetic techniques. Its capacity to provide direct anatomical visualization and controlled pharmacological delivery embodies the core principles of contemporary precision medicine. The clinical significance of UGRA is particularly evident in its dual capacity to mitigate central nervous system complications through reduced neuroinflammatory responses and to facilitate substantial opioid minimization while maintaining effective analgesia. To fully optimize implementation and outcomes, subsequent initiatives must prioritize technological refinement, the establishment of standardized proficiency-based training pathways, and the execution of rigorously designed multicenter clinical trials. Through these advancements, UGRA will continue to evolve as an indispensable component of geriatric perioperative medicine, ultimately enhancing care quality and recovery outcomes for the expanding population of aging surgical patients.

## Data Availability

The original contributions presented in the study are included in the article/supplementary material, further inquiries can be directed to the corresponding author.
